# Preparation of Magnetic Biochar Derived from Spent Mushroom Substrate and Its Adsorption and Regeneration Performance for NH_4_^+^ and PO_4_^3−^

**DOI:** 10.3390/molecules31111949

**Published:** 2026-06-04

**Authors:** Junlin Zhai, Wende Wang, Jiaxiang Tang, Bin Liu, Zebing Xing

**Affiliations:** College of Agricultural Engineering, Shanxi Agricultural University, Jinzhong 030801, China; 15563505149@163.com (J.Z.); 13563797288@163.com (W.W.); 13754823878@163.com (J.T.); 20233058@stu.sxau.edu.cn (B.L.)

**Keywords:** mushroom substrate, magnetic biochar, adsorption, ammonium, phosphate

## Abstract

Nitrogen and phosphorus are the primary pollutants responsible for eutrophication in water bodies, and their effective removal is crucial for water environmental protection. Biochar, owing to its porous structure and surface functional groups, exhibits excellent adsorption performance for nitrogen and phosphorus, which can be significantly enhanced through metal modification. In this study, magnetic biochar (MBC) was prepared from spent mushroom substrate via FeCl_3_ impregnation and microwave pyrolysis, and its adsorption performance for NH_4_^+^ and PO_4_^3−^ was systematically evaluated. The physicochemical properties of MBC were characterized using scanning electron microscopy, thermogravimetric analysis, specific surface area and pore structure analysis, vibrating sample magnetometry, and Fourier transform infrared spectroscopy. The results showed that the saturated magnetization of MBC was 7.86 emu/g, the specific surface area was 37 m^2^/g, and the material exhibited a mesoporous structure with high thermal stability. The adsorption process followed pseudo-second-order kinetics, and the mechanisms involved electrostatic interactions, surface complexation, and pore filling. Isotherm studies indicated that the maximum adsorption capacities of MBC for NH_4_^+^ and PO_4_^3−^ were 16.25 mg/g and 14.99 mg/g, respectively. Thermodynamic analysis revealed that the adsorption of NH_4_^+^ was exothermic, whereas that of PO_4_^3−^ was endothermic. Furthermore, MBC maintained an adsorption efficiency of up to 93% after ten adsorption–desorption cycles, demonstrating excellent reusability.

## 1. Introduction

Nitrogen and phosphorus are major pollutants in wastewater treatment processes, significantly impacting aquatic ecosystems. Their sources include agriculture, industry, and domestic wastewater, with ammonia nitrogen and total phosphorus being the predominant forms [1]. In water bodies, these nutrients can lead to eutrophication, excessive algal growth, algal blooms, and deteriorated water quality and biodiversity [2]. Therefore, effective removal of nitrogen and phosphorus is crucial for maintaining aquatic environment health.

Biochar exhibits excellent adsorption performance for nitrogen and phosphorus compounds due to its porous and aromatic structure, which endows it with broad application prospects in the ecological management and restoration of eutrophic water bodies [3]. The physiochemical properties of biochar vary significantly depending on the type of feedstock and the modification methods employed, thereby affecting its adsorption efficiency for nitrogen and phosphorus compounds in aquatic environments. Numerous studies have focused on incorporating metals such as calcium [4], aluminum [5], iron, manganese [6], and magnesium [7] to produce metal (oxyhydro)oxides as the primary active adsorbents, thereby enhancing its removal capabilities. Studies have shown that compared to unmodified biochar, calcium-modified biochar can achieve up to a 75-fold increase in phosphorus adsorption capacity, followed by magnesium-modified (69-fold) and iron-modified (17-fold) biochars, respectively [7]. Recent research further indicates that Ca/N co-doped biochar exhibits a maximum phosphorus adsorption capacity of up to 145.47 mg/g [8]. Moreover, compared to unmodified corn straw biochar, magnesium-loaded biochar enhances the adsorption capacity for NH_4_^+^ and PO_4_^3−^ by 8-fold and 9-fold, respectively [9].

Magnetic biochar, as a functional environmental adsorbent, demonstrates significant application potential in water pollution remediation. Its primary advantage lies in the ingenious integration of a high specific surface area and abundant surface chemical properties characteristic of porous carbon materials with the unique magnetic responsiveness of magnetic nanoparticles (such as Fe_3_O_4_ or γ-Fe_2_O_3_). This not only confers exceptional adsorption capacity and selectivity for various pollutants (including heavy metal ions and organic micropollutants), but more importantly addresses the technical bottlenecks of conventional powdered biochar, such as difficulties in solid–liquid separation and the risk of secondary pollution, thereby enabling rapid, efficient, and low-energy recovery and recycling through the application of an external magnetic field [10]. Magnetic biochar preparation methods are flexible and diverse. In industrial production, based on the differences in magnetization processes, they can be classified into several types, including the impregnation pyrolysis method, co-precipitation method, reduction deposition method, and hydrothermal method [11]. Compared with the co-precipitation method (which may suffer from pore blockage due to loading), the reduction deposition method (which involves complex processes), or the hydrothermal method (which requires advanced equipment and has limited production capacity), the impregnation pyrolysis method directly integrates the magnetic precursor with the raw biomass. This one-step pyrolysis process simultaneously achieves the carbonization of the biochar and the in situ formation of magnetic particles, offering a streamlined process route, relatively lower energy consumption, and the advantage of utilizing existing pyrolysis equipment, thereby facilitating scale-up production [12]. Compared to conventional impregnation pyrolysis, which relies on external conduction heating and easily results in uneven heat distribution and migration-agglomeration of magnetic particles, the impregnation-microwave pyrolysis technique leverages the volumetric heating characteristics of microwaves to rapidly and uniformly heat the biochar precursor from the inside out. This not only dramatically shortens the pyrolysis time and reduces energy consumption, but more importantly, it effectively locks in the initial distribution sites of the impregnated iron salts, thereby generating magnetic nanoparticles that are smaller in size and more uniformly distributed. Ultimately, this leads to the production of high-performance magnetic biochar with stronger magnetic responsiveness and better-preserved adsorption pore structures. However, current research on the combined preparation of magnetic biochar via impregnation and microwave pyrolysis remains limited.

According to statistics, by 2023, the total production of edible mushrooms in China reached 43.3604 million tons, marking a 2.69% year-on-year increase. Based on the estimate that approximately 5 kg of discarded mushroom substrate by-product is generated per kilogram of fresh edible mushrooms, the overall production of this discarded substrate has exceeded 200 million tons [13]. Given its high production volume, extensive sources, and complex composition, improper disposal of the discarded substrate not only leads to resource wastage but also results in a series of environmental pollution issues. Consequently, the safe disposal and resource utilization of discarded mushroom substrate have become prominent topics in current research [14]. Discarded mushroom substrate has excellent adsorption performance and can effectively remove heavy metal ions such as Cd^2+^ and Cr^6+^ from water and soil, as well as organic pollutants [15,16]. Jin et al. prepared 16 biochar adsorbents from four mushroom substrate residues and investigated the effects of pyrolysis temperature and raw material composition on their adsorption performance for Cu^2+^. The results showed that biochar from mushroom substrate residues efficiently removed Cu^2+^ by pyrolysis at high temperatures (500–600 °C) [17]. Ahmad et al. prepared biochar from Mg-Fe-modified mushroom substrate to remove phosphate from water. The results showed that the prepared biochar had a high specific surface area and pore volume, as well as rich functional groups necessary for phosphate removal, and could efficiently remove phosphate [18].

Based on the analysis above, this study aims to (i) investigate both the potential and practical viability of producing MBC via microwave pyrolysis under high temperatures using discarded edible mushroom cultivation substrates; (ii) evaluate the adsorption capacity for nitrogen and phosphorus of the magnetic biochar derived from spent mushroom substrates under various operating conditions; and (iii) elucidate the adsorption isotherms and kinetic models while clarifying the corresponding adsorption mechanisms. This research offers a novel strategy for a novel approach to harnessing the scientific potential of mushroom substrates and provides significant reference for environmental science research.

## 2. Results and Discussion

### 2.1. Characterization of MBC

**Figure 1:**
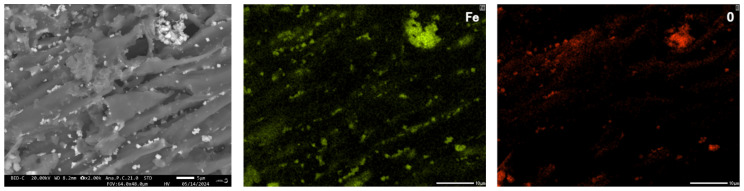
SEM images of MBC and the corresponding EDS spectra.

**Figure 2:**
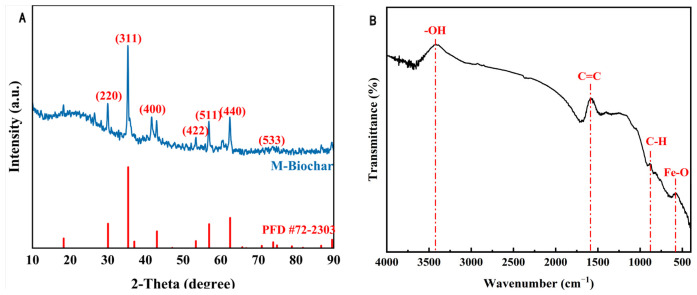
(**A**) XRD and (**B**) FTIR patterns of MBC.

SEM images show that Fe_3_O_4_ particles are uniformly distributed on the surface and within pores of the magnetic biochar (Figure 1), bestowing the biochar with strong ferromagnetism. This facilitates rapid, efficient, and low-cost solid–liquid separation after adsorption [19]. The EDS spectrum of the MBC surface shows that Fe_3_O_4_ nanoparticles are covered by abundant oxygen and iron on the biochar surface. XRD analysis of the biochar (Figure 2A) shows a crystalline phase of magnetite, with characteristic peaks corresponding to the (220), (311), (400), (422), (511), and (440) reflections [20]. The XRD results confirm successful introduction of iron ions onto the biochar surface, forming magnetite. The FTIR spectrum of MBC (Figure 2B) reveals key surface functional groups. The broad peak at 3409 cm^−1^ is attributed to O-H stretching vibration, originating from residual structural water; surface hydroxyl groups (–OH) inherent to the lignocellulosic framework of the mushroom substrate; and potentially from hydroxyl groups on the surface of hydrated iron oxides [21]. The peak at 1578 cm^−1^ corresponds to the C=C stretching vibration of aromatic rings, which is a hallmark of the carbonized structure [22]. This group primarily derives from the cyclization and aromatization of lignin and cellulose components in the feedstock during high-temperature microwave pyrolysis, forming a stable, graphitic-like carbon matrix. The peak at 875 cm^−1^, assigned to C–H bending, likely arises from the out-of-plane deformation of aromatic C–H bonds, indicating the persistence of some hydrogenated aromatic structures even after pyrolysis [23]. Critically, the distinct absorption band at 573 cm^−1^ confirms the Fe–O stretching vibration, providing direct spectroscopic evidence for the successful incorporation of magnetite (Fe_3_O_4_) crystals onto the biochar surface [24]. This Fe–O functionality, formed in situ from the impregnated FeCl_3_ precursor during pyrolysis, is the primary source of the material’s superparamagnetic property, which is essential for its post-adsorption recovery and reusability.

**Figure 3:**
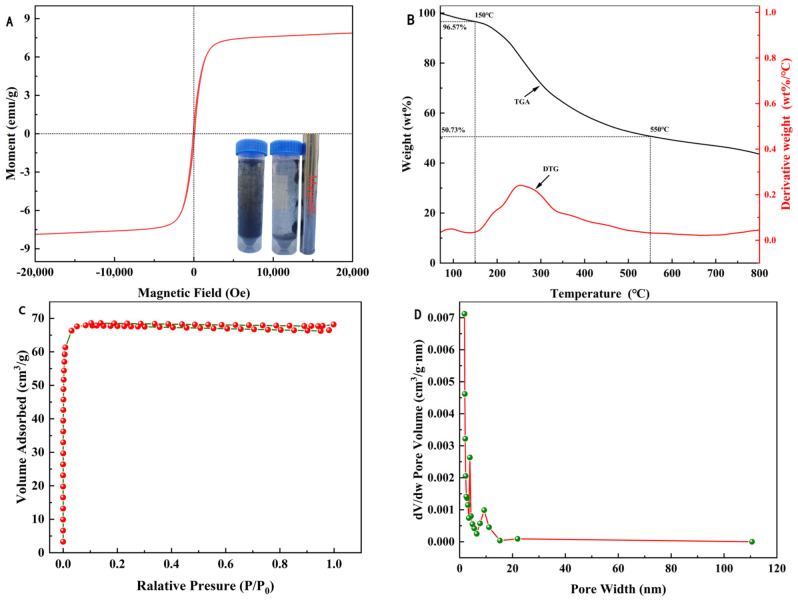
(**A**) Magnetic hysteresis loop, (**B**) thermogravimetric curve, (**C**) N_2_ adsorption–desorption isotherm, and (**D**) pore volume and pore size distribution curve of MBC.

As shown in Figure 3A, the magnetization curve indicates a saturation magnetization of 7.86 emu/g and an almost negligible remanence, demonstrating typical superparamagnetic behavior, confirming the successful synthesis of magnetic biochar [25]. Thermogravimetric analysis (Figure 3B) revealed that the biochar underwent three distinct weight loss stages between 65 and 800 °C. At lower temperatures, MBC exhibited minimal mass loss, primarily attributed to the evaporation of residual free water within the biochar. However, starting from 150 °C, MBC demonstrated a significant weight loss process, particularly in the range of 150–550 °C, with a weight loss rate reaching 49.27%, mainly due to the thermal decomposition of lignin and cellulose. At higher temperatures, the condensed and refractory organic carbon fractions underwent thermal decomposition, yet the weight loss rate markedly decreased, indicating excellent thermal stability [26]. In addition, the nitrogen adsorption–desorption isotherm (Figure 3C) displays a typical type IV isotherm with an H3-type hysteresis loop, and a distinct step is observed in the medium-to-high pressure range (P/P_0_ ≈ 0.4–0.8), indicating the presence of mesoporous structures in the sample [27]. Pore size analysis (Figure 3D) shows that the biochar primarily consists of micropores and small mesopores, with a minor amount of macropores. Finally, the nitrogen adsorption/desorption test determined that the specific surface area of the biochar is 37 m^2^/g, suggesting that it possesses a certain adsorption capacity.

### 2.2. Mechanism of Biochar Formation

The edible mushroom cultivation substrate primarily contains organic compounds such as lignocellulose, chitin, and polysaccharides. Its porous structure and surface functional groups (e.g., –OH, –COOH) provide binding sites for iron ions. During impregnation, Fe^3+^ diffuses thoroughly into the interior of the biomass through mechanisms such as ion exchange, complexation, and physical adsorption [28]. During the drying process, FeCl_3_ hydrolyzes into FeOOH or Fe(OH)_3_, which covers the surface of the biomass and forms a precursor complex. During pyrolysis, the reducing gases (CO, H_2_) produced from biomass decomposition gradually reduce Fe^3+^ to form Fe_3_O_4_ [29,30].

### 2.3. Adsorption Capacity of Biochar

As seen in Figure S1, within pH 2 to 12, biochar’s adsorption of NH_4_^+^ increases with pH, whereas its adsorption of PO_4_^3−^ decreases. From an electrostatic standpoint, biochar interacts with both NH_4_^+^ and PO_4_^3−^. The zero-point charge of biochar, determined by the pH drift method (Figure S2), is 3.165, signifying that the surface becomes negatively charged when the solution pH exceeds this value. This change enhances NH_4_^+^ adsorption and inhibits PO_4_^3−^ adsorption. Thus, the electrostatic adsorption mechanism is crucial in NH_4_^+^ and PO_4_^3−^ adsorption by biochar.

**Figure 4:**
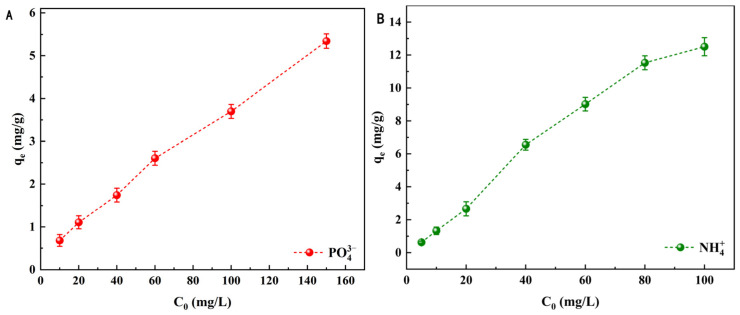
The effect of initial concentrations of (**A**) PO_4_^3−^ and (**B**) NH_4_^+^ on MBC adsorption capacity.

**Figure 5:**
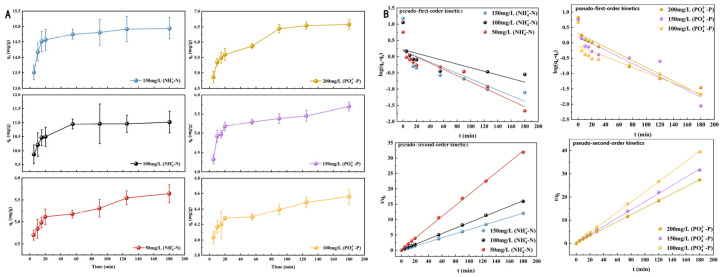
(**A**) Effect of adsorption time on the adsorption capacity of biochar and (**B**) pseudo-first-order kinetics and pseudo-second-order kinetics.

To evaluate the impact of initial NH_4_^+^ and PO_4_^3−^ concentrations on biochar’s adsorption efficiency, adsorption experiments were conducted by adding biochar to NH_4_^+^ solutions with concentrations of 5–100 mg/L and PO_4_^3−^ solutions with concentrations of 10–150 mg/L. As seen in Figure 4, as the initial concentrations of NH_4_^+^ and PO_4_^3−^ increased, the adsorption capacity of biochar also increased until reaching equilibrium (NH_4_^+^ increased from 0.63 mg/g to 12.50 mg/g; PO_4_^3−^ increased from 0.68 mg/g to 5.34 mg/g). This is mainly due to the increased collision frequency between biochar and the adsorbates caused by higher solution concentrations [31]. Additionally, Figure 5A shows the influence of adsorption time on the adsorption of NH_4_^+^ and PO_4_^3−^. The adsorption efficiency increases dramatically within the first 20 min. After 20 min, the removal rate continues to rise, although the rate of increase slows down gradually, and beyond 120 min, the change in adsorption efficiency becomes negligible, essentially reaching equilibrium. This is because after 20 min, the adsorption sites are gradually occupied by the adsorbates, leading to a decrease in the number of effective adsorption sites.

With increasing temperature, the adsorption capacity for NH_4_^+^ decreases gradually, while that for PO_4_^3−^ increases (Figure S3). The positive ΔG^0^ indicates the non-spontaneity of NH_4_^+^ adsorption on MBC [32] (Table 1). The negative ΔH^0^ and ΔS^0^ indicate the exothermic process and decreased disorder at the biochar–solution interface [33]. For PO_4_^3−^ adsorption on MBC, the positive ΔG^0^ indicates a non-spontaneous process; however, the positive ΔH^0^ reveals an endothermic process, and the negative ΔS^0^ reflects increased disorder at the biochar–solution interface. Additionally, the optimal adsorbent dosages for NH_4_^+^ and PO_4_^3−^ are 2 mg/mL and 4 mg/mL, respectively (Figure S4).

### 2.4. Adsorption Kinetics

The adsorption kinetics of NH_4_^+^ and PO_4_^3–^ on biochar were analyzed using pseudo-first order and pseudo-second order models. The fitted curves are shown in Figure 4B and the fitting results are presented in Table 2. The results indicate that the pseudo-second order model has a significantly higher R^2^ value than the pseudo-first order model, and the equilibrium adsorption capacity (qe,cal) calculated from the pseudo-second order model closely matches the experimental value (qe,exp). Therefore, the pseudo-second order kinetic model more accurately describes the adsorption process of NH_4_^+^ and PO_4_^3–^ on biochar, implying that chemisorption is the predominant mechanism [34].

**Figure 6:**
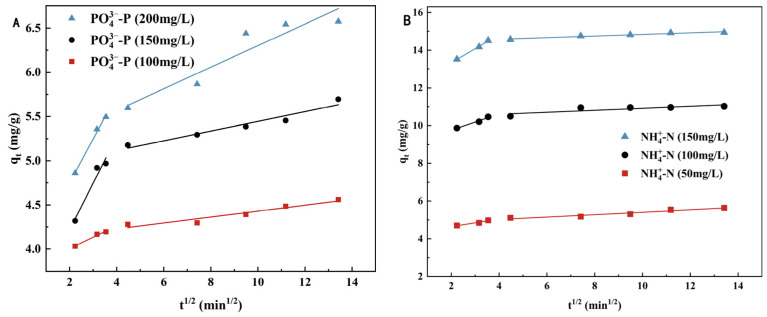
Intraparticle diffusion model (**A**) PO_4_^3−^, (**B**) NH_4_^+^.

In addition, this study employed the Weber-Morris model to examine whether the adsorption rate is primarily controlled by external diffusion, internal diffusion, or both. The biochar adsorption process can be divided into two stages (Figure 6). In the first stage, a steep slope is observed, which is mainly attributable to the boundary layer effect, whereby NH_4_^+^ and PO_4_^3–^ ions are rapidly adsorbed onto the biochar boundary layer through film diffusion, leading to a significant increase in adsorption capacity. In the second stage, these ions diffuse from the macropores on the biochar surface into its internal pores, resulting in a gradual decrease in the adsorption rate. The results, as shown in Table 3, indicate that k_1_ is much greater than k_2_, suggesting that the rate of external mass transfer significantly exceeds that of intraparticle diffusion. This implies that while the adsorption process is controlled by surface chemical reactions, it is also markedly influenced by intraparticle diffusion.

**Figure 7:**
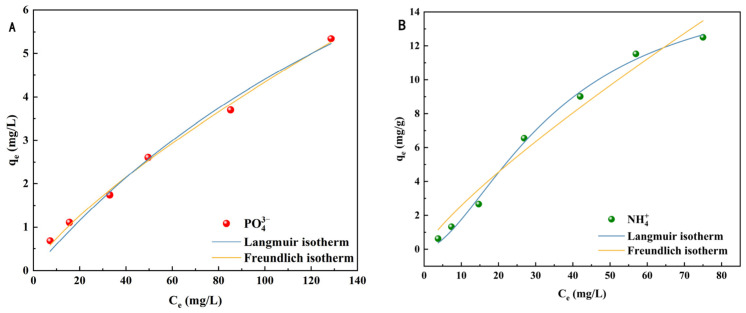
Adsorption equilibrium isotherms of MBC adsorption (**A**) PO_4_^3−^, (**B**) NH_4_^+^.

Figure 7 shows the fitted curve of the Langmuir, Freundlich and Dubinin–Astakhov adsorption isotherm models, and the related fitting parameters are listed in Table 4. Both isotherm models exhibit good agreement with the adsorption data (R^2^ > 0.95). For PO_4_^3−^, the R^2^ value of the Langmuir model (R^2^ = 0.9891) is lower than that of the Freundlich model (R^2^ = 0.99625), indicating that the primary adsorption of PO_4_^3−^ occurs on a surface with uniform energy rather than through a heterogeneous multilayer adsorption process. Thus, the Freundlich isotherm model can more accurately describe the adsorption of PO_4_^3−^ on biochar compared to the Langmuir model. For NH_4_^+^, the R^2^ value of the Langmuir model (R^2^ = 0.9964) is higher than that of the Freundlich model (R^2^ = 0.9721), suggesting that the adsorption process takes place primarily on surface sites with relatively uniform energy in a monolayer manner. As shown in Table 4, the 1/n values for PO_4_^3−^ and NH_4_^+^ are 0.76769 and 0.82178, respectively, both falling between 0.1 and 1, which demonstrates that they are readily adsorbed onto biochar.

### 2.5. The Reusability of Biochar

**Figure 8:**
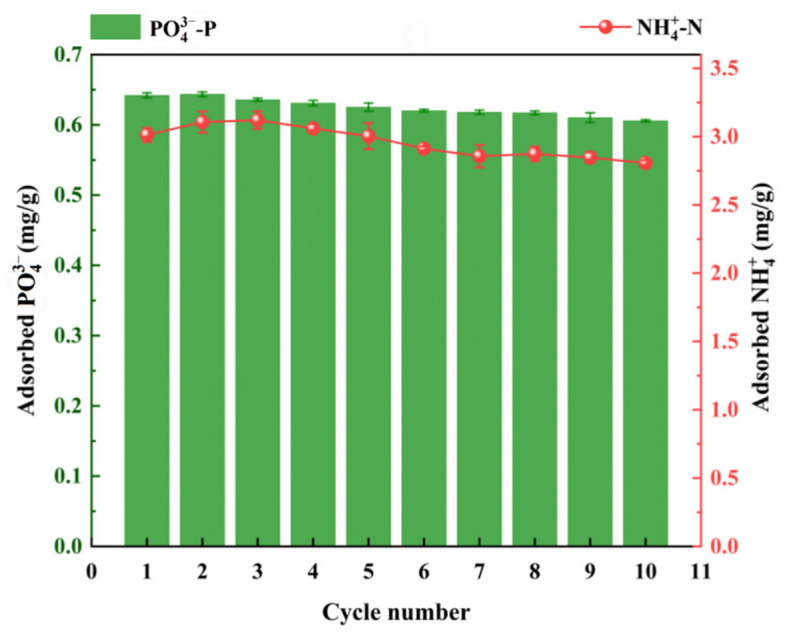
Cyclic regeneration experiments on the adsorption of NH_4_^+^ and PO_4_^3−^ onto MBC.

The reusability of the adsorbent is an important indicator of its performance. The recovery of magnetically modified biochar, after it has reached adsorption saturation, is evaluated by examining its adsorption capacity over multiple regeneration cycles. As shown in Figure 8, after undergoing 10 adsorption–desorption cycles, the biochar maintained a high adsorption capacity. Under the same experimental conditions, the adsorption efficiencies for PO_4_^3–^ and NH_4_^+^ decreased by only 5.65% and 6.84%, respectively, indicating that the synthesized biochar adsorbent exhibits excellent cyclic stability.

## 3. Materials and Methods

### 3.1. Materials

Discarded mushroom cultivation substrate (Pleurotus ostreatus substrate) was obtained from the spent mushroom cultivation base at Shanxi Agricultural University. The substrate was dried at 105 °C for 24 h. After drying, it was milled to pass through a 40–150 mesh sieve (0.1–0.45 mm) for subsequent use. Ferric chloride hexahydrate (FeCl_3_·6H_2_O), potassium dihydrogen phosphate (KH_2_PO_4_), and ammonium chloride (NH_4_Cl), all of analytical reagent grade, were purchased from Maclin Company (Shanghai, China).

### 3.2. Synthesis and Characterization of MBC

Ten grams of the prepared waste edible fungus substrate and 4.821 g of FeCl_3_·6H_2_O were added to a beaker containing 50 mL of distilled water, stirred, and soaked for 48 h. After soaking, the iron-loaded spent mushroom substrate obtained by filtration was dried at 105 °C for 24 h. The dried sample was then placed in a silicon nitride boat and subsequently loaded into a microwave pyrolysis furnace (CY-OY1100C-S; Hunan Changyi Microwave Technology Co., Ltd., Changsha, China). Next, N_2_ (200 mL/min) was injected into the furnace to establish an oxygen-deficient pyrolysis environment. The furnace was heated from room temperature to 600 °C at a power of 700 W, maintained at the target temperature for 1 h, and then allowed to cool naturally to room temperature. Finally, the biochar sample was collected. The morphological features and surface functional groups of the synthesized MBC adsorbent were analyzed using scanning electron microscopy (SEM, JSM-IT800, JEOL, Tokyo, Japan) with a scanning resolution of 20 kV and magnifications of 2 k and Fourier-transform infrared spectroscopy (FTIR-1500, Josvok, Tianjin, China) at a scanning resolution of 4 cm^−1^. The thermal properties of the biochar were characterized by thermogravimetric analysis (STA449F3-QMS403C, Netzsch, Selb, Germany) with a heating rate of 10 K/min. The specific surface area and pore structure were determined by nitrogen adsorption–desorption measurements (3H-2000PS1, BSD, Shenzhen, China) under degassing conditions of 300 °C for 180 min. At 25 °C, within a magnetic field range of −20,000 to 20,000 Oe, the hysteresis loop of the biochar was recorded using a vibrating sample magnetometer (VSM, 8600 Series, Lake Shore Cryotronics, Inc., Woburn, MA, USA). The ultraviolet-visible absorption spectrum was measured using a UV-vis spectrophotometer (UV-2600i, Shimadzu, Beijing, China), and the pHPZC of the biochar sample was determined using the pH drift method [35]. According to this method, the pH of a 0.01 mol/L sodium chloride solution was adjusted to nine gradients ranging from 2 to 10 using 0.1 mol/L NaOH and 0.1 mol/L HCl. Then, 30 mg of MBC was added to 10 mL of the above NaCl solution, which was placed in a shaker. After setting the conditions at 25 °C, 250 rpm for 8 h, the final pH of each solution was measured. The pH_PZC_ value was determined by plotting the final pH against the initial pH.

### 3.3. Adsorption Experiment

In the adsorption experiments, reserve solutions containing 1000 mg/L of NH_4_^+^–N and PO_4_^3−^–P were prepared using NH_4_Cl and KH_2_PO_4_, respectively. Subsequently, adsorption solutions of varying concentrations were obtained by diluting the corresponding reserve solutions. The adsorption experiments were conducted in a constant-temperature (25 °C) shaking chamber at 200 rpm.

To determine the optimal adsorbent dosage, 50 mL of NH_4_^+^ and PO_4_^3–^ solutions were mixed with biochar at concentrations ranging from 0.25 to 6 mg/mL. The effect of pH on the adsorption capacity was investigated by varying the pH from 2 to 12. To assess the influence of the initial solution concentration on adsorption efficiency, adsorption experiments were conducted by adding 0.1 g and 0.2 g of biochar (Figure S4) to 50 mL of NH_4_^+^ solution (5–100 mg/L) and PO_4_^3–^ solution (10–150 mg/L), respectively. The adsorption efficiency of biochar for solutions with different concentrations was examined over a 3-h period, and further analysis was carried out on its adsorption efficiency at temperatures ranging from 20 to 45 °C. In the adsorption experiments, the suspension was filtered using a 0.45 µm syringe filter to obtain the sample solution, and the residual concentrations of NH_4_^+^ and PO_4_^3–^ were measured using the Nessler reagent spectrophotometric method (HJ 535-2009) and the ammonium molybdate spectrophotometric method (GB 11893-89), respectively [36,37,38,39].

The adsorption capacity of biochar is calculated using Equation (1), as follows:

(1) $$q_{e}=\frac{(C_{0}-C_{\mathrm{eq}})V}{m}$$

q_e_ (mg/g) represents the adsorption capacity of the biochar for NH_4_^+^ and PO_4_^3−^ at equilibrium, and C_0_ (mg/L) and C_eq_ (mg/L) denote the initial and equilibrium solution concentrations, respectively. V (L) is the volume of the adsorption solution, and m (g) is the amount of biochar used in the adsorption process.

In order to gain a deeper understanding of the adsorption kinetics of NH_4_^+^ and PO_4_^3−^ on biochar, a linear fitting of the experimental data was performed using pseudo-first-order kinetics, pseudo-second-order kinetics, the Langmuir and Freundlich isotherms, as well as a particle diffusion kinetics model.

### 3.4. Reusability Assessment

Under conditions of 25 °C, a solution pH of 7.0, and the optimal biochar dosage, 50 mL of a simulated wastewater containing 20 mg/L NH_4_^+^ and 5 mg/L PO_4_^3−^ was subjected to adsorption for 24 h [40]. Afterward, the mixture was filtered to collect the biochar, which was then added to a solution consisting of 50 mL of 0.1 mol/L NaOH and 1 mol/L NaCl for shaking desorption for 2 h. The biochar was filtered, rinsed with distilled water until neutral, and dried at a constant temperature of 60 °C. This process was repeated, and the adsorption capacity and removal efficiency for NH_4_^+^ and PO_4_^3−^ were calculated after each adsorption experiment. To evaluate the stability and reusability of the biochar, ten regeneration cycles were conducted.

## 4. Conclusions

In this study, waste edible mushroom substrate was successfully utilized as a raw material to prepare magnetic biochar with superparamagnetic properties, a mesoporous structure, and high thermal stability via FeCl_3_ impregnation and microwave pyrolysis. The material exhibited excellent adsorption performance for NH_4_^+^ and PO_4_^3−^ in aqueous solutions, with maximum adsorption capacities of 16.25 mg/g and 14.99 mg/g, respectively. The adsorption process followed a pseudo-second-order kinetic model, indicating that chemisorption predominated. The isothermal adsorption behaviors conformed to the Langmuir and Freundlich models, respectively, with the adsorption mechanism involving electrostatic interactions, surface complexation, and pore filling. The solution pH and temperature significantly influenced the adsorption performance, and thermodynamic analysis revealed that the adsorption of NH_4_^+^ was exothermic, whereas that of PO_4_^3−^ was endothermic. After ten adsorption–desorption cycles, the removal efficiency of the magnetic biochar for both pollutants decreased by less than 7%, demonstrating excellent stability and reusability. This study provides an effective approach for the resource utilization of waste edible mushroom substrates, and the developed magnetic biochar holds promising potential for the remediation of nitrogen- and phosphorus-polluted water bodies.

## Figures and Tables

**Table 1:** Thermodynamic parameters of MBC adsorption.

Temperature/K	PO_4_^3−^			Temperature/K	NH_4_^+^		
	ΔG^0^ (KJ mol^−1^)	ΔH^0^ (KJ mol^−1^)	ΔS^0^ (J mol K^−1^)		ΔG^0^ (KJ mol^−1^)	ΔH^0^ (KJ mol^−1^)	ΔS^0^ (J mol K^−1^)
293	7.90	17.49	−33.30	293	4.82	−7.19	−40.92
298	7.49			298	5.00		
303	7.19			303	5.21		
308	7.24			308	5.30		
313	7.21			313	5.69		

**Table 2:** Adsorption kinetic parameters of MBC adsorption.

C_0_ (mg/L)		qe,exp (mg/g)	Pseudo-First-Order-Kinetics			Pseudo-Second-Order-Kinetics		
			qe,cal (mg/g)	k_1_ (min^−1^)	R^2^	qe,cal (mg/g)	k_2_ (g min^−1^ min^−1^)	R^2^
PO_4_^3−^	100	4.58	0.79	0.01997	0.7508	4.56	0.18450	0.9998
	150	5.70	1.59	0.02517	0.8104	5.67	0.09362	0.9992
	200	6.61	2.01	0.02554	0.8986	6.66	0.06115	0.9996
NH_4_^+^	50	5.66	1.52	0.02211	0.8401	5.63	0.08724	0.9991
	100	11.30	1.64	0.0128	0.4934	11.24	0.07829	0.9996
	150	15.01	1.50	0.0199	0.6248	14.96	0.13473	0.9999

**Table 3:** Parameters of the intraparticle diffusion model for MBC adsorption.

C_0_ (mg/L)		First Stage			Second Stage		
		k_1_ (mg g^−1^h^−1/2^)	c_1_ (mg/g)	R^2^	k_2_ (mg g^−1^h^−1/2^)	c_2_ (mg/g)	R^2^
PO_4_^3−^	100	0.12974	3.74625	0.9828	0.03388	4.09201	0.9254
	150	0.52618	3.16789	0.9524	0.05480	4.89814	0.9388
	200	0.49423	3.76454	0.9939	0.12217	5.08043	0.8916
NH_4_^+^	50	0.20118	4.24512	0.9513	0.06361	4.77230	0.9237
	100	0.44158	8.86431	0.9760	0.05221	10.39704	0.7011
	150	0.75481	11.81653	0.9975	0.04288	14.40082	0.9488

**Table 4:** Adsorption parameters of the Langmuir and Freundlich isotherm models for MBC adsorption.

	Langmuir			Freundlich		
	K_L_	q_m_	R^2^	K_F_	n	R^2^
PO_4_^3−^	0.00416	14.99115	0.9891	0.12645	1.30261	0.9962
NH_4_^+^	0.00257	16.24649	0.9964	0.388	1.21687	0.9721

## Data Availability

The datasets generated during and/or analyzed during the current study are available from the corresponding author on reasonable request.

## Supplementary Materials

The following supporting information can be downloaded at: https://www.mdpi.com/article/10.3390/molecules31111949/s1, Figure S1. Effect of pH on the adsorption capacity of MBC: (A) PO_4_^3−^, (B) NH^4+^; Figure S2. pHPZC titration measurement of MBC; Figure S3. Effect of temperature on the adsorption capacity of MBC: (A) PO_4_^3−^, (B) NH^4+^; Figure S4. Effect of biochar dosage on the adsorption capacity of MBC: (A) PO_4_^3−^, (B) NH^4+^ (solution concentration of 60 mg/L).
