# Supplementary material for: Preparation of Magnetic Biochar Derived from Spent Mushroom Substrate and Its Adsorption and Regeneration Performance for NH4+ and PO43−
Source: Molecules. 2026 Jun 4;31(11):1949. doi: 10.3390/molecules31111949 (PMC13257525; doi:10.3390/molecules31111949)
Supplement: Supplementary file 1 [file molecules-31-01949-s001.zip › molecules-4307418-supplementary.pdf]

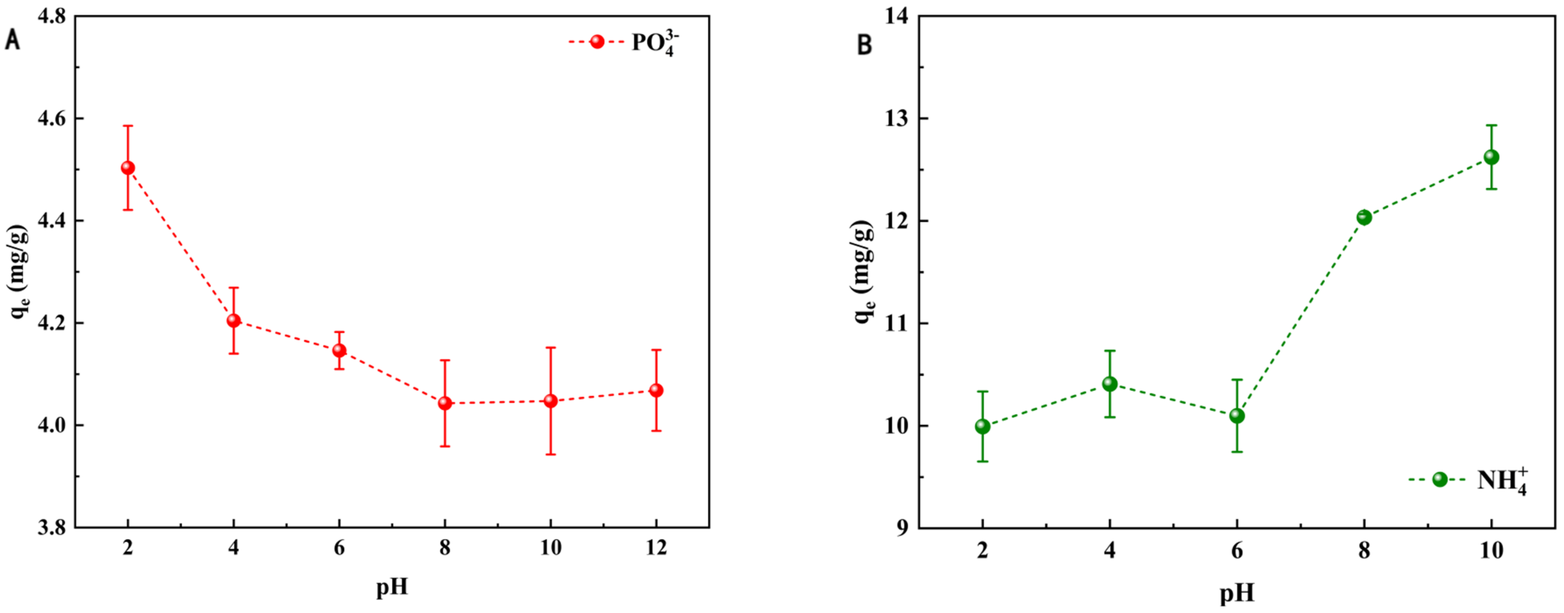

Figure S1. Effect of pH on the adsorption capacity of MBC: (A) PO_4^{3-}, (B) NH_4^+.

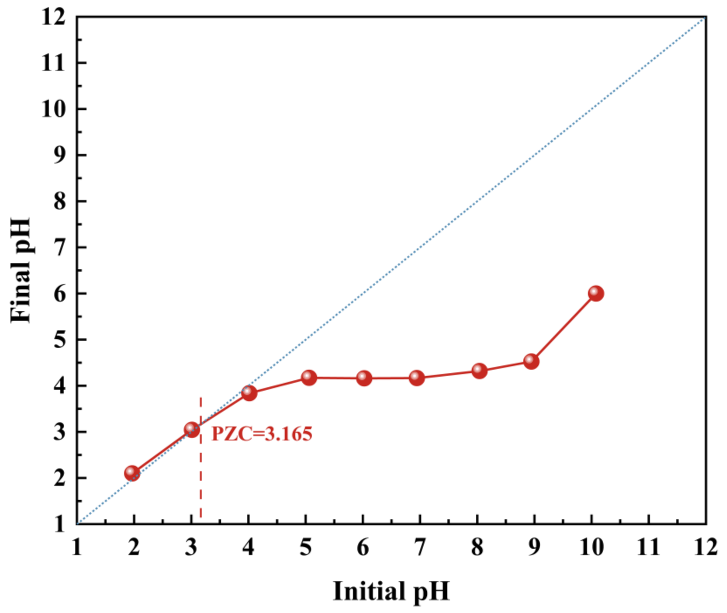

Figure S2. pH_{PZC} titration measurement of MBC.

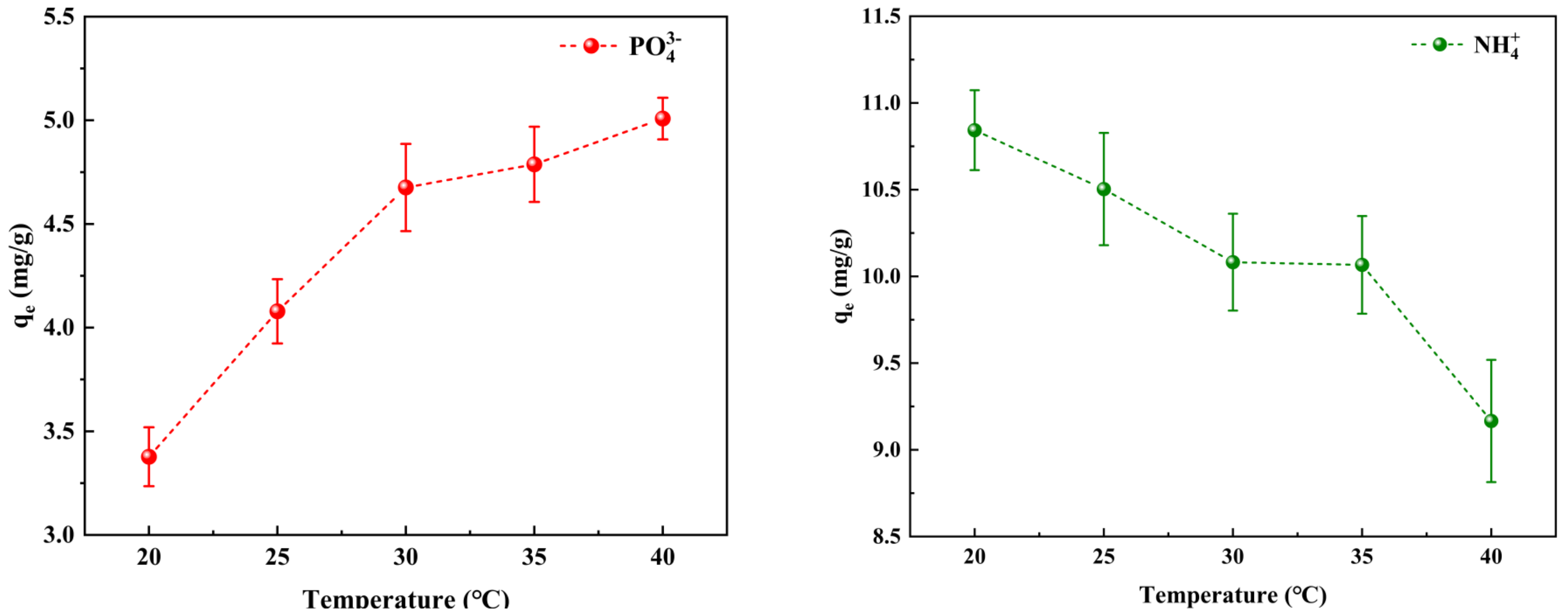

Figure S3. Effect of temperature on the adsorption capacity of MBC: (A) PO_4^{3-}, (B) NH_4^+.

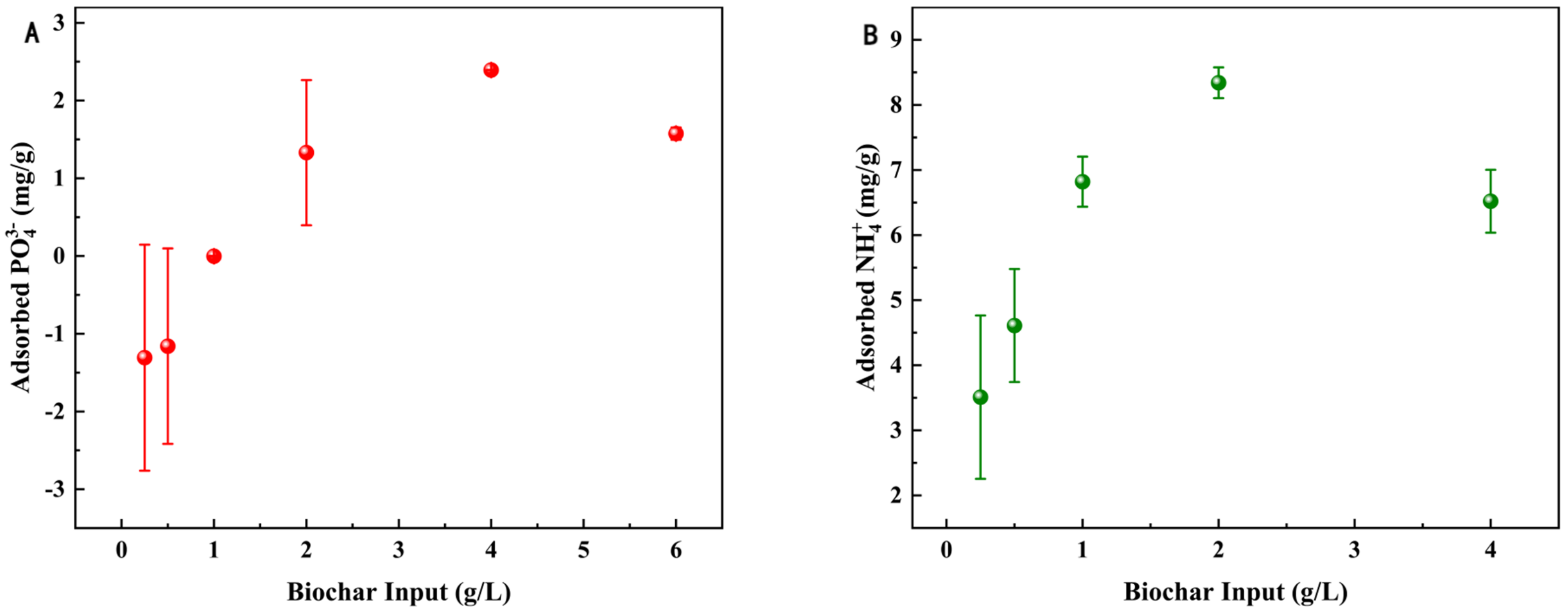

Figure S4. Effect of biochar dosage on the adsorption capacity of MBC: (A) PO_4^{3-}, (B) NH_4^+ (solution concentration of 60 mg/L).
